# Long-Term Engagement With a Physical Activity App Among Gynecologic Cancer Survivors in the LETSGO Trial: 12-Month Prospective, Multicenter, Quasi-Experimental Study

**DOI:** 10.2196/84838

**Published:** 2026-08-13

**Authors:** Sindre Herskedal Fosstveit, Ingvild Vistad, Mette Skorstad, Sveinung Berntsen

**Affiliations:** 1 Department of Sport Science and Physical Education, Faculty of Health and Sport Sciences, University of Agder, Universitetsveien 25, Kristiansand, 4630, Norway, 47 94822211; 2 Research Unit, Sorlandet Hospital, Kristiansand, Norway; 3 Department of Gynecology and Obstetrics, Sorlandet Hospital, Kristiansand, Norway; 4 Institute of Clinical Medicine, University of Oslo, Oslo, Norway; 5 Medical Department, Clinical Institute II, University of Bergen, Bergen, Norway

**Keywords:** survivorship care, mobile health, mHealth, eHealth, mobile app, step count, engagement, adherence, self-management, nurse-led follow-up, real-world evidence

## Abstract

**Background:**

Physical activity (PA) alleviates many treatment-related side effects in gynecologic cancer survivors, yet long-term PA levels remain low. Mobile health interventions can support self-management and increase PA levels; however, evidence from real-world, year-long engagement with smartphone apps in this population is still limited.

**Objective:**

The aim of this study is to describe 12-month user engagement with the PA component of a smartphone app implemented within a partially nurse-led routine follow-up in a real-world cohort of gynecologic cancer survivors.

**Methods:**

This descriptive study analyzed server-generated log data from the intervention arm of the prospective, multicenter, quasi-experimental LETSGO (Lifestyle and Empowerment Techniques in Survivorship of Gynecologic Oncology) trial (NCT04122235). Between December 2019 and July 2022, 378 cancer survivors (ovarian, endometrial, cervical, vulvar, or vaginal cancer) from 5 Norwegian hospitals were enrolled in the intervention arm and were offered the app plus a Garmin Vivofit 4 activity tracker alongside standard consultations. Primary outcomes for this study were (1) weekly PA registrations (objective step counts from the activity tracker and self-reported PAs) and (2) temporal patterns of step logging during each cancer survivor’s first 52 weeks postenrollment. Secondary analyses compared baseline characteristics of app users (≥2 wk of PA logging) and nonusers.

**Results:**

Of 378 eligible participants (mean age 63, SD 13 y; BMI 28.5, SD 6.4 kg/m²), 272 (72%) logged at least 2 weeks of PA, and 225 (60%) synchronized objective step data. Mean daily steps were 5657 (SD 2799; median 5533, IQR 3499‐7498). Step tracking dominated app use (mean 21, SD 16 logged wk), followed by self-reported walking (mean 23, SD 19 wk) and resistance training (mean 15, SD 16 wk). Weekly step-logger counts fell around study weeks 16, 32, and 44, but rebounded by 30 to 50 participants within 3 weeks, indicating episodic rather than permanent disengagement. App users were younger (mean difference –6.7 y, 95% CI –9.6 to –3.9; *P*<.001), more often employed (*χ*²_1_=12.7; *P*<.001), and more likely to have higher education (*χ*²_2_=13.6; *P*<.001) than nonusers. Tumor type and treatment modality were not associated with engagement.

**Conclusions:**

In a routine-care setting, nearly three-quarters of gynecologic cancer survivors engaged repeatedly with an app-supported PA module over 12 months, although mean PA levels were modest and participation was more prevalent among younger, employed, and more highly educated participants. Engagement followed an ebb-and-flow pattern, suggesting that built-in re-engagement prompts and equity-focused onboarding are needed to sustain and broaden participation. These findings support the feasibility of blended mobile health follow-up while highlighting the importance of adaptive strategies to promote long-term PA adherence and bridge the digital divide among cancer survivors.

## Introduction

Survivorship rates after gynecologic cancers are rising globally, leading to a growing number of women living with long-term treatment sequelae [1]. Gynecologic cancer survivors frequently experience treatment-related late effects that can significantly impact their quality of life, with fatigue, neuropathy, lymphedema, sexual dysfunction, cognitive impairment, anxiety, and depression being among the most commonly reported [2-8].

Regular physical activity (PA) has been shown to alleviate many late effects, providing both psychological and physiological benefits [9]. In addition, PA is associated with improved health-related quality of life and a reduced risk of cancer-related morbidity, making it a crucial component of survivorship care [10,11]. Consistent with this, objectively measured PA has recently been associated with a lower risk of cardiovascular disease in cancer survivors [12]. In recent years, increasing emphasis has been placed on promoting self-management in cancer rehabilitation, with a focus on fostering a healthy lifestyle [13], of which PA is a central element. Despite these recognized benefits and the growing focus on self-managed survivorship care, PA engagement among gynecologic cancer survivors remains low, often due to treatment-related side effects, fear of injury, and limited referral pathways to exercise professionals [14-16]. This persists despite contemporary practice guidance recommending routine brief PA promotion and formal referral within multidisciplinary oncology care [17].

Mobile health (mHealth) interventions, including smartphone apps and wearable PA trackers, have emerged as promising tools for promoting PA among cancer survivors [18]. These technologies facilitate real-time feedback, goal setting, and self-monitoring, which may enhance motivation and adherence to PA recommendations [19]. A systematic review by Khoo et al [20] found that mHealth interventions effectively increased PA levels in cancer survivors. In practice, though, these benefits depend on continued use. Engagement with mHealth typically declines after an early peak, and maintaining use over time has proved more difficult than achieving initial uptake [21,22]. The decline is rarely steady: many patients stop for a period and then start again, so looking only at average use can mask meaningful patterns of disengagement and re-engagement [23]. Even so, real-world evidence on how cancer survivors engage with these tools over the long term (≥12 mo) remains limited, particularly among gynecologic cancer survivors [18,24].

Existing evidence on mHealth interventions in populations with cancer is primarily based on controlled trials with selected, potentially more motivated populations, which may limit the generalizability of findings to broader cancer survivor groups [25]. Uptake of digital health also tends to be lower among older, less-educated, and lower-income survivors, widening a digital divide that real-world programs need to address [26]. To address this evidence-practice gap, the LETSGO (Lifestyle and Empowerment Techniques in Survivorship of Gynecologic Oncology) project was developed as a pragmatic program embedded in routine, partly nurse-led follow-up across multiple Norwegian hospitals, with app support to promote self-management among gynecologic cancer survivors [27]. A key feature of the app was its PA component, which included goal setting, PA tracking, and structured exercise programs tailored to users with varying levels of prior PA experience. Implemented without exclusions based on baseline PA levels, LETSGO enabled evaluation of real-world engagement with digital PA tools in a clinically heterogeneous cohort of gynecologic cancer survivors.

Accordingly, the aim of this study was to explore user engagement with the PA component of the LETSGO app over the first 12 months of follow-up. Specifically, we examined how participants interacted with the PA module by assessing PA logging frequency, the types of PA recorded, and patterns of objectively recorded steps over time.

## Methods

### Study Design and Setting

This study is part of the LETSGO project, a prospective, multicenter, quasi-experimental clinical cohort study (ClinicalTrials.gov identifier: NCT04122235) evaluating a follow-up program for patients with gynecologic cancer, which includes alternating nurse-led and physician-led follow-up consultations at the intervention hospitals [28]. The nurses focused on coaching techniques and were supported by a mobile app. The complete study spans 3 years, comparing the combined intervention (nurse-led consultations and a mobile app) with standard follow-up care at control hospitals [27]. Patients followed the intervention or the standard pathway according to their treatment hospital rather than through individual randomization [28].

This study provides a descriptive analysis of 1-year usage data from the PA component of the LETSGO app. Data were collected from participants in the intervention group from 5 hospitals, who had access to the LETSGO app as part of their follow-up care. Usage patterns and PA logging behavior were examined based on app-generated log data collected over each participant’s first 12 months in the study. Server log data (eg, PA registrations in the app) were collected automatically through a secure server using an encrypted connection.

### Inclusion and Exclusion Criteria

Participants were eligible if they were aged 18 years or older, were within 3 months of completing primary treatment for a histologically verified gynecologic malignancy (ovarian, endometrial, cervical, vulvar, or vaginal cancer), and had entered the survivorship follow-up phase of care.

Participants were excluded if they had disease progression at the time of enrollment, were participating in other clinical trials, required ongoing intravenous therapy treatment, or had undergone trachelectomy for cervical cancer. Any participant who experienced cancer recurrence or progression during the study was subsequently excluded and referred for appropriate treatment.

### Participant Characteristics

The 378 enrolled participants were gynecologic cancer survivors with endometrial, ovarian, cervical, vulvar, or vaginal cancer.

### Sampling Procedures

Between December 2019 and July 2022, all eligible patients entering survivorship follow-up at the 5 intervention hospitals were identified and invited consecutively by clinical staff, using information from medical records and multidisciplinary team meetings; no random selection or prespecified quota was applied. The 378 enrolled participants received access to the LETSGO app as part of their routine follow-up care.

### Sample Size, Power, and Precision

No a priori sample-size calculation was performed for this analysis; the parent LETSGO trial was powered for its primary outcome of change in patient empowerment, which required 377 participants per group [28]. As a descriptive study, this analysis included all 378 survivors enrolled in the intervention arm during the recruitment period, representing the full available population.

### Ethical Considerations

**Figure 1.**
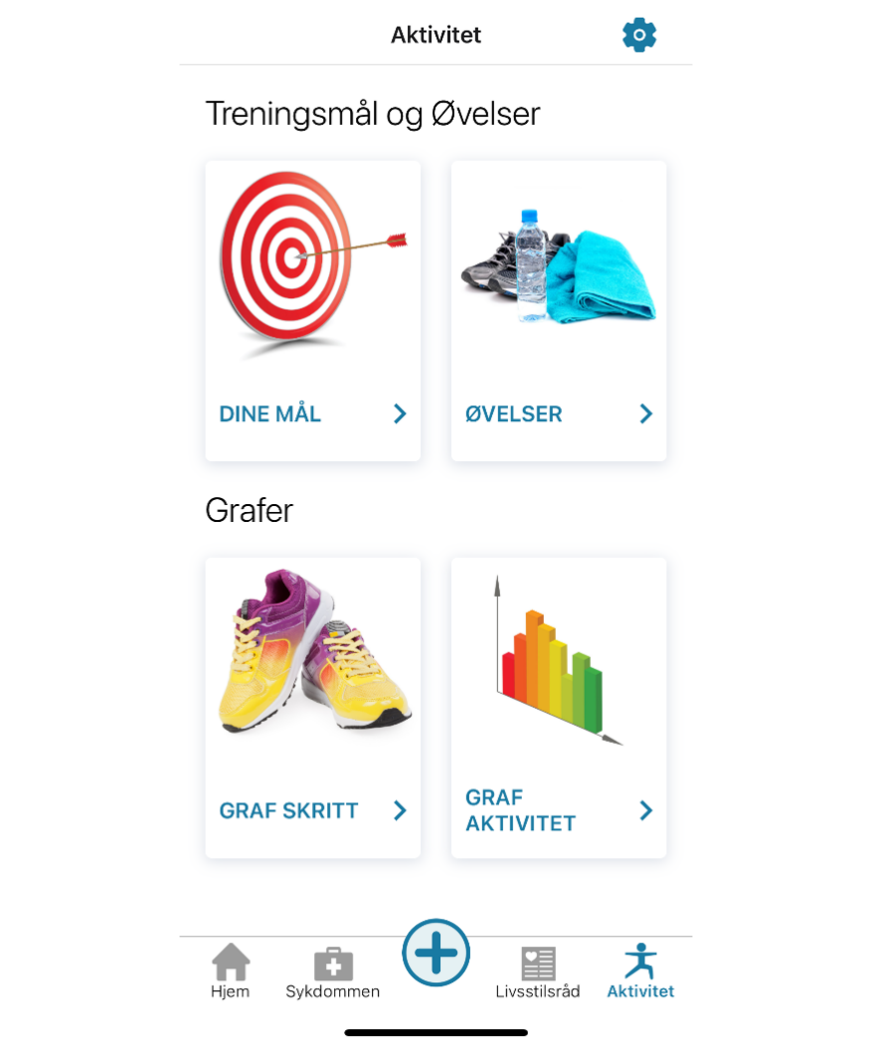
Overview of the physical activity interface of the LETSGO (Lifestyle and Empowerment Techniques in Survivorship of Gynecologic Oncology) smartphone app used by gynecologic cancer survivors. The interface includes sections for setting training goals (*Dine Mål*), accessing structured exercise programs (*Øvelser*), and monitoring activity data through graphical representations of step count (*Graf Skritt*) and logged activities (*Graf Aktivitet*).

The LETSGO study was approved by the Regional Committee of Medical Research Ethics in Norway (2019/11093) and received additional approval from the institutional review board and the data protection officer at each study site. The study was conducted in accordance with Norwegian data protection regulations, and all participants provided written informed consent. Participants were informed of their right to withdraw from the study at any time without consequences for their further treatment. All data were deidentified prior to analysis, with participants assigned a unique study identifier; no directly identifiable personal information was included in the analytic dataset. No financial compensation was provided to participants for their involvement in the study. No identifiable participants or users appear in any figure or the supplementary material in Multimedia Appendix 1; Figure 1 shows only the app interface.

### The LETSGO Intervention

A detailed description of the intervention hospitals and their roles in the LETSGO trial is provided in the protocol paper by Vistad et al [27]. In this study, only aspects relevant to the implementation of the digital follow-up program and the PA component are presented.

Throughout the follow-up period, participants alternated between nurse-led and physician-led consultations, with nurses conducting every second scheduled consultation. The nurse-led consultations incorporated motivational strategies to promote PA engagement and support participants in managing late effects of cancer treatment. The program’s intervention model draws on self-determination theory [29], which posits that sustained behavior change is promoted when 3 basic psychological needs are supported: autonomy, competence, and relatedness.

At their first nurse-led consultation, participants were introduced to the LETSGO app with several features, including structured information on PA, goal-setting features, and progress tracking. The app was supplemented by a Garmin activity tracker (Garmin Vivofit 4; Garmin Ltd), which enabled users to track their step counts and synchronize PA data with the app. During this consultation, nurses assessed participants’ prior PA levels and collaboratively set individualized PA goals tailored to their motivation and perceived barriers. Nurses assisted participants in downloading the app and pairing their Garmin Vivofit 4 activity tracker with the app during this initial consultation.

At follow-up consultations, nurses reviewed each participant’s app-logged PA data together with the participant, discussed progress toward individualized goals, and adjusted targets as needed. All study nurses had completed a 2-day intensive training course covering PA counseling, individualized goal setting, autonomy-supportive communication, and motivational interviewing techniques, reinforced by a mandatory e-learning program. Nurses applied an autonomy-supportive communication approach inspired by motivational interviewing [30], emphasizing participant empowerment and self-efficacy in PA adherence. Within the self-determination theory framework, autonomy was supported through this communication style and through participant-led goal setting; competence was fostered through individualized, graded exercise prescriptions and self-monitoring of progress via the app; and relatedness was maintained through the ongoing nurse-led consultations that provided a consistent relational context for PA support. Participants without smartphones (n=35) were provided with printed material containing the same PA guidance.

### PA Component of the App

The PA component of the LETSGO app was designed to support gynecologic cancer survivors in maintaining or increasing their PA levels during follow-up care. The app, available on smartphones and tablets, provided participants with structured exercise programs, goal-setting functionality, and self-monitoring tools to facilitate engagement in PA.

The PA goals and exercises section included 2 features: “your goals” and “exercises” (Figure 1). In the goal-setting module, participants recorded their weekly PA goals. The exercise module included structured resistance and endurance training programs, accompanied by written instructions and video demonstrations, suitable for both beginner and advanced levels. Detailed descriptions of the app’s training content, including resistance and endurance training modules, are provided in Multimedia Appendix 1.

The activity graphs section included a “step graph” that displayed step-count data and an “activity graph” that showed the frequency and distribution of different logged PAs (Figure 1).

The PA component of the LETSGO app incorporated several behavior change techniques (BCTs) from the BCT Taxonomy v1 [31]. Participants engaged in self-monitoring of behavior by reviewing weekly step counts and logging PA frequency through in-app graphs. Goal-setting (behavior) was supported through individual PA targets set collaboratively with the nurse at each consultation and revisited at follow-up. Graded task difficulty was embedded in the structured resistance and endurance exercise programs. Prompts and cues were delivered via push notifications, encouraging weekly activity logging.

### Measures and Covariates

The primary outcome of this study was participant engagement with the PA component of the LETSGO app over each participant’s first 12 months in the study. For descriptive analyses, an active week was defined as a study week with at least one logged PA registration, including tracker-synchronized step data and/or self-reported PA. Weekly activity was used as the unit of analysis to describe the frequency of app interaction over time.

In addition to overall engagement, the study examined the types of PAs registered in the app, including steps, jogging, cycling, resistance training, yoga or Pilates, swimming, group classes, and other self-reported activities. Step registrations were objectively measured using a Garmin Vivofit 4 activity tracker, while all other PAs were self-reported by participants. For step-based analyses, Garmin Vivofit 4 data were available in the LETSGO server log as weekly synchronized registrations. For each such week, the server provided a precomputed average daily step variable, defined as the total number of steps recorded during that calendar week divided by 7. Thus, the denominator for weekly average daily steps was always 7 calendar days within a synchronized week.

Secondary outcomes included differences in demographic characteristics between participants who engaged with the app (logged at least 2 weeks of PA app usage) and those who did not, as well as temporal patterns in step-logging frequency and the total number of daily steps recorded over the 12-month period. This threshold was chosen pragmatically to distinguish repeated use from no use or a single, isolated week of use.

Baseline covariates considered as potential correlates of engagement were age, body weight, BMI, educational level, employment status, tumor type, and treatment modality. Age (from the date of birth), tumor type, and treatment modality were obtained from the patients’ medical records, and educational level and employment status were obtained from baseline questionnaires [27,28]; BMI was calculated from weight and height (kilograms per square meter).

### Data Analysis

All statistical analyses were conducted using SPSS (version 29; IBM Corp), and figures were created using GraphPad Prism (version 8.0.1; GraphPad Software). Descriptive statistics were used to summarize demographic and clinical characteristics. The normality of continuous variables was assessed using histograms and measures of skewness and kurtosis. Independent *t* tests (2-tailed) were performed for normally distributed variables, while the Mann-Whitney *U* test was used for nonnormally distributed variables. Associations between categorical variables were examined using the Pearson chi-squared test. Statistical significance was set at *P*<.05 for all analyses. The methods were structured and reported in accordance with the APA Journal Article Reporting Standards for Quantitative Research (JARS-Quant) [32]. This study was reported in accordance with the STROBE (Strengthening the Reporting of Observational Studies in Epidemiology) guideline for cohort studies (Checklist 1). Missing baseline data were quantified; the missing-data mechanism was assessed with the Little test for data missing completely at random, and a multiple-imputation sensitivity analysis (*m*=20, chained equations, pooled with Rubin rules) was performed for the variables with missing data.

## Results

### Study Participants

**Figure 2.**
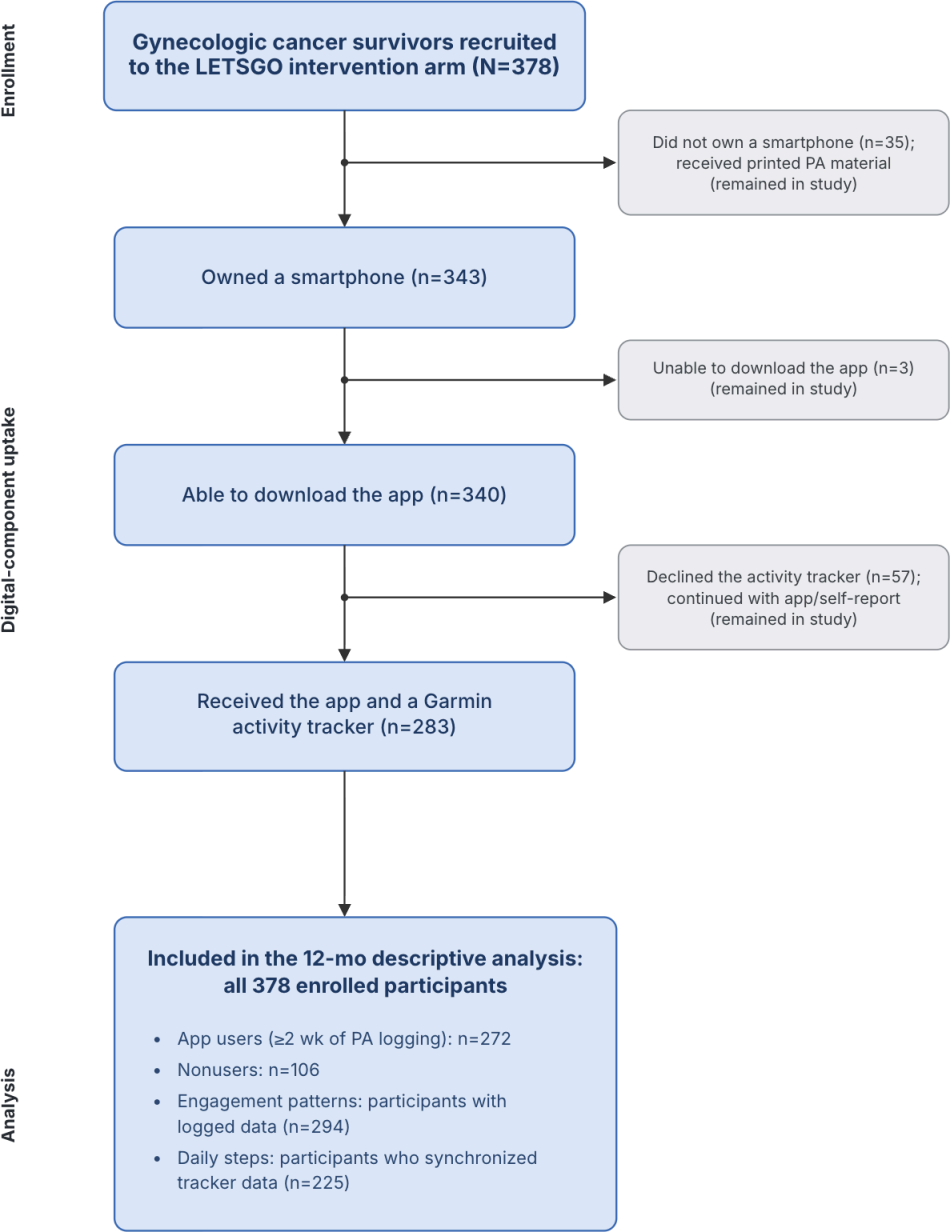
Participant flow through the LETSGO (Lifestyle and Empowerment Techniques in Survivorship of Gynecologic Oncology) intervention arm. The chart shows enrollment, uptake of digital components (smartphone, app, and Garmin activity tracker), and analysis. Counts beside the main pathway indicate the number of participants who did not take up a given component; all 378 enrolled participants were included in the 12-month descriptive analysis. PA: physical activity.

Figure 2 displays participant flow through the digital pathway of the LETSGO intervention. Of the 378 recruited participants, 343 (90.7%) owned a smartphone, and 340 (89.9%) were able to download the app. A total of 283 (74.9%) received both the app and a Garmin activity tracker.

The baseline demographic and clinical characteristics, overall, and by app-engagement group, are summarized in Table 1.

Participants who engaged with the PA component of the app were significantly younger than those who did not (mean difference –6.7 y, 95% CI –9.6 to –3.9; *t*_376_=–4.6; *P*<.001). No significant differences were observed for body weight (mean difference 0.2 kg, 95% CI −4.5 to 4.8; *P*=.94) or BMI (mean difference −0.3 kg/m², 95% CI −1.9 to 1.3; *P*=.73).

There were significant associations between educational level (*χ*²_2_=13.6; *P*<.001) and engagement with the app for PA, and between employment status (*χ*²_1_=12.7; *P*<.001) and engagement with the app for PA. There were no significant associations of cancer diagnosis or treatment modality with engagement with the app for PA.

Missing baseline data for educational level, employment status, and weight or BMI are summarized in Table 1; all other variables (age, tumor type, and treatment modality) were complete.

**Table 1.** Baseline demographic and clinical characteristics of gynecologic cancer survivors in the LETSGO^a^ intervention arm, overall, and by app-engagement group^b^.

Variable	Total (N=378)	App user (n=272)	Non–app user (n=106)	*P* value
Age (y), mean (SD)	63 (13)	61 (12)	68 (13)	<.001
BMI (kg/m^2^), mean (SD)	28.5 (6.4)	28.4 (6.5)	28.7 (6.3)	.73
Missing, n	41	14	27	
Body weight (kg), mean (SD)	79 (19)	79 (19)	79 (17)	.94
Tumor type, n (%)				.27
Endometrial	221 (59)	154 (57)	67 (63)	
Ovarian	92 (25)	73 (27)	19 (18)	
Cervical	58 (15)	41 (15)	17 (16)	
Vulvar or vaginal	7 (2)	4 (2)	3 (3)	
Treatment modality, n (%)				.31
Surgery only	213 (56)	147 (54)	66 (62)	
Chemotherapy and radiotherapy	19 (5)	16 (6)	3 (3)	
Surgery and chemotherapy	121 (32)	93 (34)	28 (26)	
Other combinations^c^	25 (7)	16 (6)	9 (9)	
Employment^d^, n (%)				<.001
Yes	120 (35)	105 (40)	15 (19)	
No	219 (65)	154 (60)	65 (81)	
Missing, n	39	13	26	
Education level^d^, n (%)				<.001
Primary	50 (15)	28 (11)	22 (28)	
Secondary	156 (46)	125 (48)	31 (39)	
Medium or higher	133 (39)	106 (41)	27 (34)	
Missing, n	39	13	26	

a LETSGO: Lifestyle and Empowerment Techniques in Survivorship of Gynecologic Oncology.

b Percentages may not sum to 100 due to rounding.

c Includes chemotherapy only or any other combination of treatments, including hormonal treatment.

d Not all participants reported their employment and education levels; thus, the total is lower than the total number of participants. App user: a participant who logged physical activity (PA) in the app in ≥2 distinct wk during their first 52-wk postenrollment (counting objective step synchronizations and/or self-reported PA registrations). Non–app user: a participant with <2 wk of PA logging in the app (ie, 0 or 1 wk).

### PA Logging and Volume

Table 2 presents the number of participants who recorded different PAs in the LETSGO app on a weekly basis, along with the mean number of PA registrations per participant, where each registration represents 1 week of recorded activity.

Step tracking was the most frequently used feature, recorded by 225 (60%) participants, with an average of 21 (SD 16) weeks of registration. Walking (n=53; mean 23, SD 19 wk) and resistance training (n=39; mean 15, SD 16 wk) were the most commonly reported self-logged activities. Other types of PA, such as bicycling, yoga, and swimming, were logged by fewer participants, with an average of 10 to 30 participants.

**Table 2.** Engagement with objective step tracking and self-reported physical activity types in the LETSGO^a^ app over 52 weeks, among gynecologic cancer survivors (N=378)^b^.

Modality	Participants, n (%)	Weeks logged per participant	
		Mean (SD)	Median (IQR)
Objective (wearable-synchronized)			
Steps	225 (60)	21 (16)	18 (7‐35)
Self-reported			
Walking	53 (14)	23 (19)	21 (3‐41)
Resistance training	39 (10)	15 (16)	7 (2‐21)
Bicycling	30 (8)	13 (14)	8 (3‐17)
Other	24 (6)	15 (17)	10 (3‐25)
Group classes	14 (4)	7 (11)	3 (2‐8)
Jogging	10 (3)	12 (11)	10 (4‐17)
Yoga or Pilates	9 (2)	12 (13)	9 (1‐17)
Swimming	9 (2)	7 (6)	6 (2‐12)

a LETSGO: Lifestyle and Empowerment Techniques in Survivorship of Gynecologic Oncology.

b Percentages are based on the total cohort (N=378). Weeks logged represents the number of distinct weeks during which a participant recorded the modality, averaged across participants who ever logged it.

Table 3 presents the weekly duration of performed PAs recorded in the LETSGO app. Participants registered an average of 5657 daily steps (SD 2799, 95% CI 5291‐6023; median 5533, IQR 3499‐7498). Walking had the highest reported average weekly duration of 172 (SD 116) minutes, followed by other self-reported activities (118, SD 80 min) and yoga or Pilates (93, SD 78 min). Resistance training, group classes, and bicycling ranged from 54 (SD 36) to 74 (SD 30) minutes per week, while jogging and swimming had the lowest average durations, at 37 (SD 20) and 40 (SD 16) minutes, respectively.

**Table 3.** Weekly physical activity volume by modality recorded in the LETSGO^a^ app over 52 weeks, among gynecologic cancer survivors (N=378)^b^.

Modality	Participants, n	Weekly dose	
		Mean (SD)	Median (IQR)
Objective, steps/d			
Steps	225	5657 (2799)	5533 (3499‐7498)
Self-reported, min/wk			
Walking	53	172 (116)	147 (92‐230)
Resistance training	39	61 (38)	52 (34‐73)
Bicycling	30	54 (36)	53 (24‐80)
Other	24	118 (80)	99 (55‐167)
Group classes	14	74 (30)	75 (52‐89)
Jogging	10	37 (20)	32 (28‐43)
Yoga or Pilates	9	93 (78)	61 (46‐99)
Swimming	9	40 (16)	35 (34‐47)

a LETSGO: Lifestyle and Empowerment Techniques in Survivorship of Gynecologic Oncology.

b n is the number of participants who logged that modality at least once over 52 weeks (for steps, the number of participants who synchronized step data ≥1 time). The weekly dose for self-reported modalities is the average number of minutes per week during weeks when that modality was logged, averaged across participants who ever logged it. Steps are the participant-level mean daily steps across weeks with synchronized data. All values reflect weeks in which physical activity was recorded and are not averaged across the entire 52-week follow-up.

### Engagement Patterns Over Time

**Figure 3.**
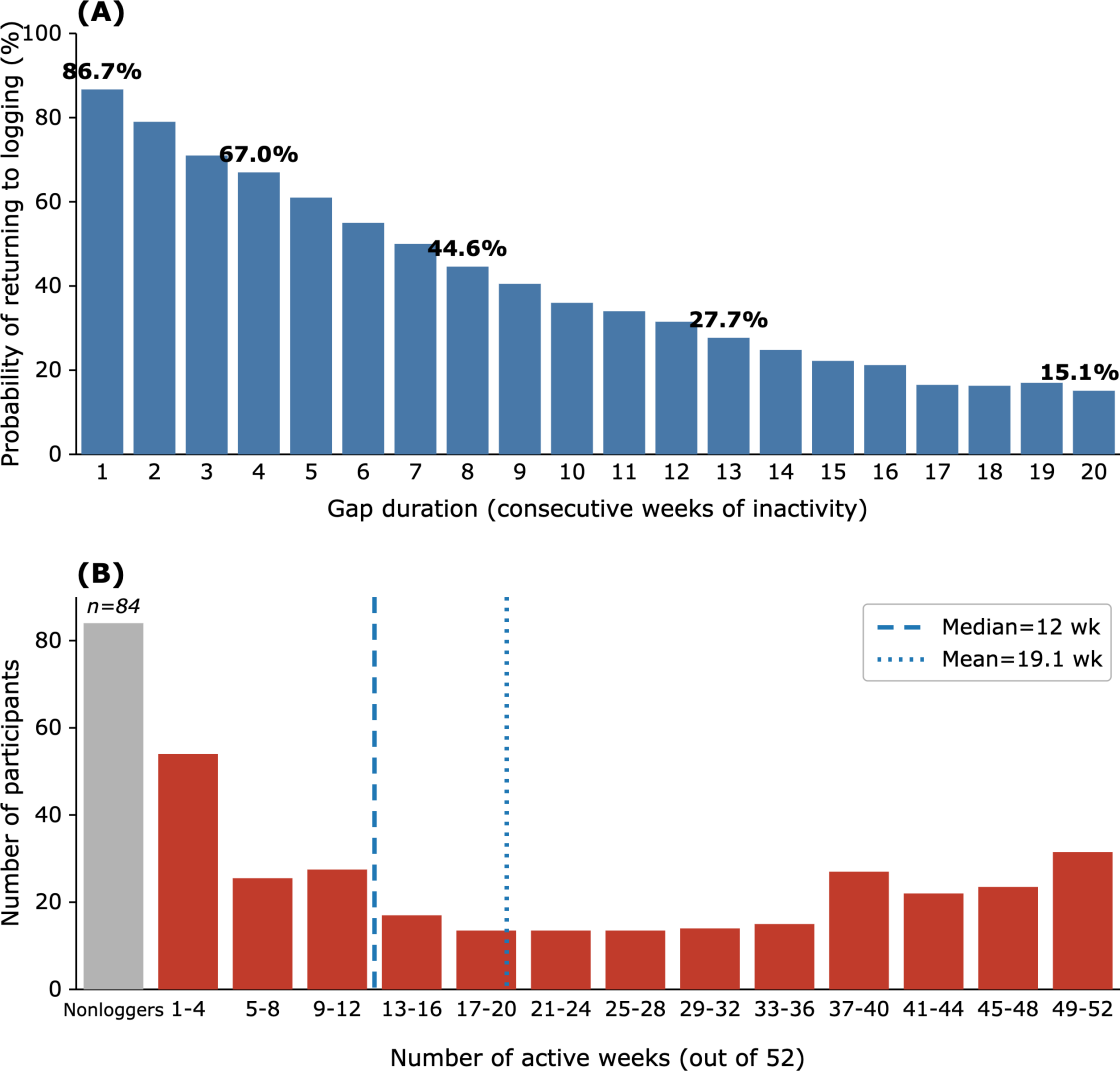
Episodic engagement with the physical activity component of the LETSGO (Lifestyle and Empowerment Techniques in Survivorship of Gynecologic Oncology) app over the first 12 months (52 wk) among gynecologic cancer survivors (N=378). (A) Probability of returning to activity logging after an inactivity gap of a given duration (n=294 participants with logged data). Participants who experienced a gap were classified as having “returned” if at least 1 additional log registration followed the gap, or as “not returned” if the gap extended to the end of the 52-week follow-up. (B) Distribution of the total number of active logging weeks (out of 52) across all 378 enrolled participants. The gray bar represents the 84 participants who never logged any activity. Red bars show the distribution among the 294 participants with logged data in 4-week intervals. Dashed and dotted lines indicate the median (12, IQR 1-38 wk) and mean (19.1, SD 18.5 wk) for all 378 participants, respectively.

Among all 378 enrolled participants, the median number of active logging weeks was 12 (IQR 1‐38; mean 19.1, SD 18.5) out of 52 available weeks. The distribution was bimodal, with concentrations at both the low end (0‐4 active wk, n=138) and the high end (41‐52 active wk, n=76; Figure 3B). Among the 294 participants who logged at least one activity registration, the median was 23.5 (IQR 8‐41) active weeks, and only 8 (3%) logged every week without interruption.

Re-engagement after inactivity gaps was common. After gaps of 1 or more weeks, 86.7% (248/286) of participants returned to logging. Return probability remained above 50% for gaps up to 7 weeks and was 27.7% (33/119) for gaps of 13 or more weeks (Figure 3A).

**Figure 4.**
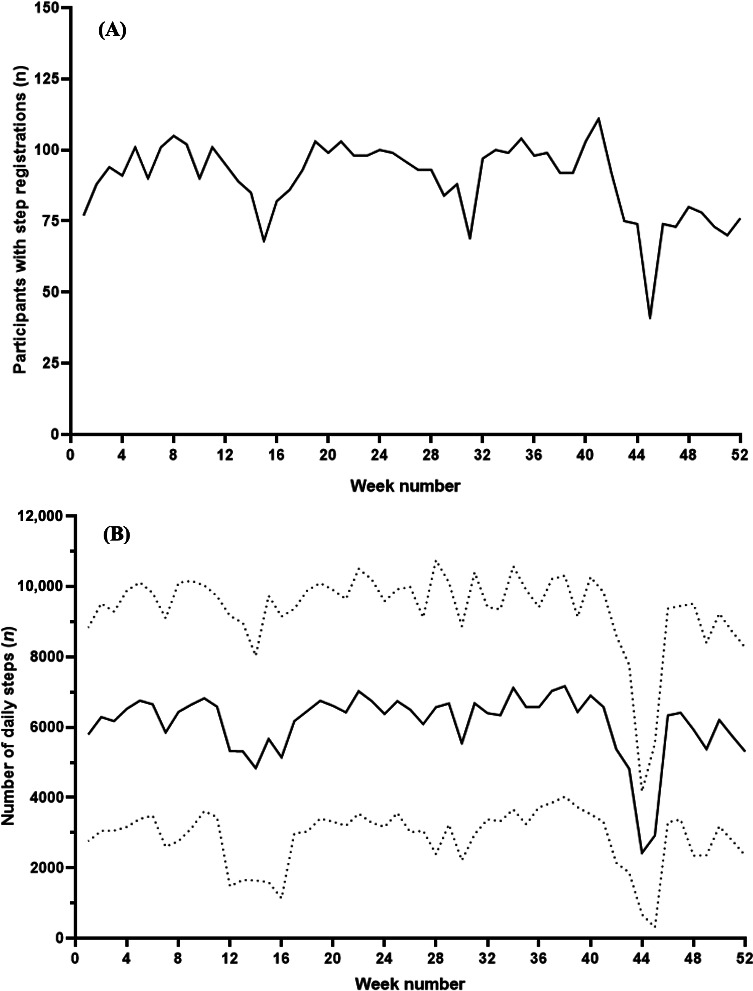
Weekly objective step engagement over the first 12 months (52 wk) among gynecologic cancer survivors in the LETSGO (Lifestyle and Empowerment Techniques in Survivorship of Gynecologic Oncology) intervention arm. (A) Number of participants registering step data each week. (B) Weekly mean daily steps. (B) The solid line represents the weekly mean number of daily steps, and the dotted lines represent the mean and 1 SD. Across the 225 participants who synchronized step data, the overall mean was 5657 daily steps (SD=2799).

Figure 4A presents the number of participants who registered their step counts over the first 12 months of the study. Weekly registrations remained stable with fluctuations, showing declines around weeks 16, 32, and 44, followed by partial recovery.

Figure 4B displays the average number of daily steps recorded by the LETSGO app. Step counts fluctuated throughout the study period, with declines occurring during the same weeks as the drop in registrations. The lowest step counts were observed in the final quarter of the study.

### Sensitivity Analysis

The Little test did not reject the assumption that the baseline data were missing completely at random (*χ*²_4_=7.13; *P*=.13). Because missingness was nonetheless somewhat more frequent among nonusers than among app users, we performed a multiple-imputation sensitivity analysis (*m*=20 imputations; chained equations; pooled with Rubin rules); the associations between engagement and educational level and employment status remained significant in each imputed dataset and after pooling (education: combined *P*=.001; employment: *P*<.001).

## Discussion

### Principal Findings

This 12-month real-world follow-up study of 378 gynecologic cancer survivors shows that almost three-quarters of those offered the LETSGO app engaged with its PA component at least twice, and that 225 participants synchronized their objective step counts via an activity tracker. Mean daily steps remained modest (≈5700, SD 2800) and fluctuated over time, with notable dips around weeks 16, 32, and 44 before a partial rebound. App engagement was higher among younger, employed, and more highly educated participants, whereas tumor type and treatment modality were not associated with usage.

### Variability in Digital Adherence

Despite evidence that mHealth interventions can increase PA levels [20], engagement remains highly variable between individuals, across studies, and over time. A 2024 systematic review by Montalescot et al [33] highlights the complexity of adherence in digital cancer care, noting that most demographic and clinical factors, including age, sex, and tumor type, show inconsistent associations with app usage. Moreover, sustaining long-term adherence to mHealth interventions remains challenging, as documented in a recent review by Amagai et al [21], which is consistent with the “law of attrition” proposed by Eysenbach [22] for eHealth interventions.

Findings from the present LETSGO cohort sharpen this picture of heterogeneity. Of the 378 participants offered the app, 272 (72%) engaged with the PA module at least twice, and 225 (60%) synchronized tracker–derived step counts during their first 12 months of individual follow-up. Participants predominantly engaged in low-impact, ambulatory PAs, with walking accounting for the majority of all recorded sessions. Yet engagement was far from uniform: users registered a mean of 21 (SD 16) weeks of step data, but the range spanned from a single upload to more than 50 weeks. Among those who logged walking, the average duration was approximately 172 minutes per week. In contrast, muscle-strengthening activities were reported less frequently, with a mean of 15 (SD 16) logged weeks and an average of approximately 61 minutes per week, rarely meeting the recommended minimum of 2 or more sessions per week [9]. Other modalities, including cycling, yoga or Pilates, swimming, and group exercise classes, were recorded sporadically by a small subset of participants (≤30). These usage patterns may reflect both technological and behavioral factors. First, automated step-count synchronization reduces the self-monitoring burden relative to manual registration, which likely contributed to the higher adherence to step tracking observed in this study [34]. Second, resistance training is a comparatively complex modality, often requiring equipment, technique mastery, and greater self-efficacy, especially for beginners. Accordingly, cancer survivors often prefer walking, which is perceived as simple, safe, and equipment-independent [16,35].

The objectively measured mean of 5657 steps per day places the cohort in the “low-active” range [36]. This step volume is below the approximate threshold of 7000 steps per day, estimated to align with the 150 minutes of moderate-intensity PA per week [36] recommended for cancer survivors by the American College of Sports Medicine and the American Society of Clinical Oncology [9,37]. However, it is comparable to those reported in other real-world accelerometer-based studies in mixed cancer populations, which typically range from 4500 to 6000 steps per day [38,39]. Epidemiological data indicate that most of the reduction in all-cause mortality risk among cancer survivors is realized at approximately 5000 to 6000 steps per day [39]. Notably, the cohort’s mean of 5657 steps per day falls within this range, indicating that participants were likely engaging in a level of PA associated with meaningful health benefits in this population. Collectively, these findings highlight that PA logging was dominated by walking and that targeted strategies to increase resistance training frequency may be necessary to achieve the full spectrum of benefits outlined in current exercise-oncology guidelines [9,37].

### Episodic Use and Re-Engagement

Real-world data from other studies confirm that engagement often wanes and is episodic rather than continuous. In the 12-month IMPACT randomized controlled trial of a mobile PA program for young adult cancer survivors, median weekly log-ins fell from 89% (23/26) of study weeks in months 0 to 6 to 42% (11/26; IQR 2‐22 wk) during months 7 to 12, with only a subset of participants sustaining regular use [40,41]. High attrition is likewise a recurring theme in the broad review by Amagai et al [21] of 62 mHealth trials, most of which were only “partially successful” at preserving long-term engagement. The LETSGO data align with these findings: the weekly number of participants uploading step data fell at several points, most notably around study weeks 16, 32, and 44, yet recovered by 30 to 50 participants within the next 1 to 3 weeks (Figure 4). These rebounds imply that a meaningful proportion of participants who became “inactive” subsequently re-engaged, illustrating that lapses frequently reflect temporary disengagement rather than permanent dropout. This ebb-and-flow dynamic suggests that blended-care models are well positioned to support re-engagement.

This analysis quantified this re-engagement pattern directly. Among the 294 participants with any logged data, 286 experienced a gap of at least 1 week; of these, 86.7% (248/286) returned to logging after a gap of at least 1 week, and the return probability remained above 50% for gaps up to 7 consecutive weeks. Even after gaps exceeding a full follow-up quarter (≥13 wk), 27.7% of participants resumed logging (Figure 3A). The distribution of active weeks was bimodal, with clusters at both the low end (0‐4 wk, n=138) and the high end (41‐52 wk, n=76), indicating 2 distinct engagement profiles rather than a uniform decline (Figure 3B). These patterns are consistent with emerging evidence that mHealth engagement is better characterized as a dynamic, nonlinear process than as a simple trajectory of decline [23]. In a systematic review and meta-analysis of app-based chronic disease interventions, Meyerowitz-Katz et al [42] reported a pooled dropout rate of 43%, yet this binary framing of “retained versus dropped out” does not capture the cyclical re-engagement observed here. Perski et al [23] have similarly argued that engagement with digital behavior change interventions involves a dynamic interplay between usage behavior and subjective experience that fluctuates within and across individuals over time. These data lend empirical support to this conceptualization and suggest that the conventional emphasis on preventing attrition may be usefully complemented by strategies that facilitate re-engagement after inevitable lapses, such as the just-in-time adaptive interventions described by Nahum-Shani et al [43].

Because each cancer survivor’s “week 0” corresponded to their enrollment date over the study period, these drops cannot be ascribed to calendar seasons but instead indicate phase-specific motivational cycles. Consistent with the mixed evidence in the review by Montalescot et al [33], tumor type and treatment modality were not associated with app use, yet analyses identified modest socioeconomic gradients: app users were younger (by ≈6.7 y) and more likely to be employed or highly educated than nonusers (Table 1). These gradients underline the persistent digital divide that many mHealth survivorship programs must still address. The BCTs embedded in the LETSGO app—self-monitoring, goal setting, graded exercise prescription, and behavioral prompting—are consistent with engagement-sustaining mechanisms identified in systematic reviews of mHealth PA interventions in cancer survivors [44,45]. The progressive structure of these techniques may partly explain the declining-but-sustained engagement pattern observed over 12 months, as goal review and graded task progression provide ongoing behavioral reinforcement beyond the initial novelty period of app adoption.

### Implications for Intervention Development

Together, the literature and these findings converge on 3 imperatives. First, engagement trajectories must be monitored continuously, recognizing that cancer survivors’ needs and motivation ebb and flow. Second, interventions should build in flexible re-engagement mechanisms, whether automated prompts, personalized feedback, or brief human contact, to draw inactive users back at critical junctures. Third, broad eligibility alone is insufficient; programs should include targeted digital-literacy assistance, alternative low-tech access routes, and tailored outreach to ensure that socioeconomically disadvantaged cancer survivors can initiate and sustain use on equal terms with their more advantaged peers.

The observed sociodemographic gradients in app use, with older, less educated, and unemployed participants consistently less likely to engage, likely reflect intersecting barriers including lower digital literacy, reduced familiarity with wearable technology, and differential access to smartphones [46]. These findings align with broader evidence of a persistent digital divide in cancer survivorship care, where older age, lower education, and socioeconomic disadvantage are consistently associated with reduced uptake of digital health tools [46,47]. Importantly, device access alone does not guarantee meaningful engagement, as barriers to uptake and sustained use operate independently of access [47]. Future survivorship programs should incorporate equity-focused implementation strategies, such as assisted device onboarding, nurse-guided digital literacy support, simplified app interfaces, and nondigital participation pathways for those unable or unwilling to engage with mHealth tools.

### Strengths and Limitations

One of the main strengths of this study is its pragmatic, real-world implementation: LETSGO was embedded in routine nurse-led survivorship care and accepted participants regardless of baseline PA level or smartphone ownership. This yielded a large, clinically heterogeneous cohort (N=378) that mirrors the breadth of gynecologic cancer follow-up, enhancing external validity. The study also combined objectively synchronized step data with self-reported PA logs, providing complementary assessments of total PA level and mode diversity while reducing recall bias. In addition, the 12-month duration, longer than most oncology mHealth trials [18,24], made it possible to examine engagement trajectories across a whole year and to document the characteristic ebb-and-flow pattern of digital adherence. Finally, evaluating the app within a blended model of autonomy-supportive nurse consultations plus digital self-monitoring offers implementation insights that are immediately transferable to other survivorship settings.

Although the LETSGO study used a longitudinal, quasi-experimental design with matched control hospitals, this analysis focuses only on the intervention arm’s PA module; therefore, it cannot establish causal relationships between app use and changes in PA or health outcomes. Wear-time compliance was not verified; therefore, gaps in step data may reflect tracker nonwear, true inactivity, or technical issues with the device, app, or server synchronization. In addition, weekly average daily steps were derived from server-generated weekly step totals divided by 7 calendar days; therefore, average daily step counts may have been underestimated. All nonstep activities were self-reported, making their duration estimates subject to recall and social-desirability bias. Despite open eligibility, engagement remained higher among younger, employed, and better-educated cancer survivors, indicating a residual digital divide and potential selection bias. Some baseline data were missing (10%‐11% for BMI, educational level, and employment status), somewhat more often among nonusers than app users, and although the Little test did not reject the assumption that these data were missing completely at random, we used multiple imputation as sensitivity analysis, which reproduced the socioeconomic associations, indicating that the findings are robust to missing data. Phase-specific dips in engagement could not be separated from contextual factors, such as treatment milestones or holidays, because analysis weeks were aligned with individual enrollment dates. Data collection spanned from December 2019 to July 2022, overlapping substantially with the COVID-19 pandemic (Norwegian lockdowns: March 2020-September 2021); pandemic-related restrictions on movement, facilities, and clinical workflows may have affected both PA levels and app engagement in ways that cannot be disentangled from individual engagement trajectories. Finally, the study took place within the Norwegian health care system; extrapolating results to regions with different infrastructures or lower smartphone penetration should be approached with caution.

Overall, the study provides a realistic demonstration of embedding a blended mHealth intervention in routine survivorship care while also highlighting methodological and equity-related challenges that future controlled and implementation-focused research will need to address.

### Conclusions

The LETSGO program showed that a heterogeneous cohort of gynecologic cancer survivors engaged with an app-supported PA module for up to 12 months. Although mean PA levels remained modest (mean 5657, SD 2799 steps/d; low-active range) and engagement fluctuated, nearly three-quarters of the participants interacted with the PA component of the app at least twice, and 60% (n=225) synchronized their objective step data with the app. Engagement lapses were typically temporary, with recurrent dips followed by re-engagement, indicating a dynamic of ebb and flow rather than definitive dropout. App use centered on walking, whereas resistance training was less frequent, with the recommended 2 or more sessions per week. Socioeconomic gradients in use, favoring younger, employed, and more highly educated participants, highlight the persistent digital divide. Collectively, these findings confirm the feasibility of pragmatic, blended digital follow-up in survivorship care, while also signaling the need for adaptive re-engagement strategies and equity-focused implementation supports, alongside targeted content to increase adherence to resistance training.

## Supplementary material

10.2196/84838 Multimedia Appendix 1 Resistance and endurance training modules.

10.2196/84838 Checklist 1 STROBE checklist.

## Abbreviations

BCT: behavior change technique
JARS-Quant: APA Journal Article Reporting Standards for quantitative research
LETSGO: Lifestyle and Empowerment Techniques in Survivorship of Gynecologic Oncology
mHealth: mobile health
PA: physical activity
RCT: randomized controlled trial
STROBE: Strengthening the Reporting of Observational Studies in Epidemiology
