# Supplementary material for: Long-Term Engagement With a Physical Activity App Among Gynecologic Cancer Survivors in the LETSGO Trial: 12-Month Prospective, Multicenter, Quasi-Experimental Study
Source: JMIR Mhealth Uhealth. 2026 Aug 13;14:e84838. doi: 10.2196/84838 (PMC13472232; doi:10.2196/84838)
Supplement: Supplementary file 1 [file mhealth-v14-e84838-s001.docx]

**1 | SUPPLEMENTARY MATERIALS**

**1.1 | Resistance Exercise Programs**

Participants had access to two structured resistance exercise programs: one designed for home-based training and one for gym-based training.

The home-based resistance exercise program included nine exercises targeting major muscle groups. The exercises were designed to be performed using minimal equipment, requiring only a mat and resistance bands for select exercises. Each exercise consisted of three sets of 8–12 repetitions and was accompanied by a detailed movement description, including instructions on execution, targeted muscle groups, and an instructional video demonstration. The exercises included squats, walking lunges, rowing with a resistance band, standing lateral raises with a resistance band, push-ups (on knees or toes), prone back extensions, sit-ups, diagonal opposite arm and leg raises, and plank.

The gym-based resistance exercise program included machine-based and bodyweight exercises performed for three sets of 10 repetitions. Similar to the home-based program, each exercise was accompanied by a written description, targeted muscle group information, and a video demonstration. The exercises included seated leg press, seated chest press, leg extension, leg curl, seated machine row, seated shoulder press, hip thrust, sit-ups, diagonal opposite arm and leg raise, and plank.

**1.2 | Endurance Exercise Programs**

The endurance training programs in the LETSGO app were structured into beginner and advanced levels. The beginner program included activities such as walking, Nordic walking, cycling, skiing, and active play with children or grandchildren. Walking sessions started at 10–15 min with progression to longer durations and higher-intensity variations, such as inclined terrain or weighted backpack walking. Nordic walking included technique instructions for arm-leg coordination and effective pole use. Cycling sessions were structured as continuous rides of 20–30 min with recommendations for increasing duration before intensity. Skiing was presented as a full-body endurance activity with guidance on terrain adjustments, and active play included activities such as pulling a sled, playing in the snow, or hiking with a backpack.

The advanced program incorporated structured interval training sessions. Streetlight intervals consisted of running or fast walking between 2–3 streetlights, followed by a walking recovery to the next, repeated 10 times. Pyramid intervals followed a progressive structure, alternating between increasing and decreasing work durations, with rest periods set to half the duration of the effort. A typical sequence included 1-minute work (30-second rest), 2-minute work (1-minute rest), 3-minute work (1.5-minute rest), continuing up and down. Hill “sprints” involved walking or running a marked uphill distance of 100–200 meters, followed by controlled walking back down, repeated 5–10 times. Participants were instructed to warm up before these sessions and include a cooldown period afterward.
